# Supplementary material for: Direct strain correlations at the single-atom level in three-dimensional core-shell interface structures
Source: Nat Commun. 2022 Oct 10;13:5957. doi: 10.1038/s41467-022-33236-6 (PMC9551052; doi:10.1038/s41467-022-33236-6)
Supplement: Supplementary file 1 — Revised Supplementary Information [file 41467_2022_33236_MOESM1_ESM.pdf]

Supplementary Information

for

Direct strain correlations at the single-atom level in three-dimensional core-shell interface structures

Hyesung Jo[1], Dae Han Wi[2], Taegu Lee[3], Yongmin Kwon[2], Chaehwa Jeong[1], Juhyeok Lee[1], Hionsuck Baik[4], Alexander J. Pattison[5,6], Wolfgang Theis[5], Colin Ophus[6], Peter Ercius[6], Yea-Lee Lee[7], Seunghwa Ryu[3], Sang Woo Han[2,*] and Yongsoo Yang[1,*]

[1] *Department of Physics, Korea Advanced Institute of Science and Technology (KAIST), Daejeon 34141, South Korea*

[2] *Department of Chemistry, Korea Advanced Institute of Science and Technology (KAIST), Daejeon 34141, South Korea*

[3] *Department of Mechanical Engineering, Korea Advanced Institute of Science and Technology (KAIST), Daejeon 34141, South Korea*

[4] *Korea Basic Science Institute (KBSI), Seoul 02841, South Korea*

[5] *Nanoscale Physics Research Laboratory, School of Physics and Astronomy, University of Birmingham, Edgbaston, Birmingham B15 2TT, UK*

[6] *National Center for Electron Microscopy, Molecular Foundry, Lawrence Berkeley National Laboratory, Berkeley, California 94720, USA*

[7] *Chemical Data-Driven Research Center, Korea Research Institute of Chemical Technology (KRICT), Daejeon 34114, South Korea*

*Email: sangwoohan@kaist.ac.kr, yongsoo.yang@kaist.ac.kr

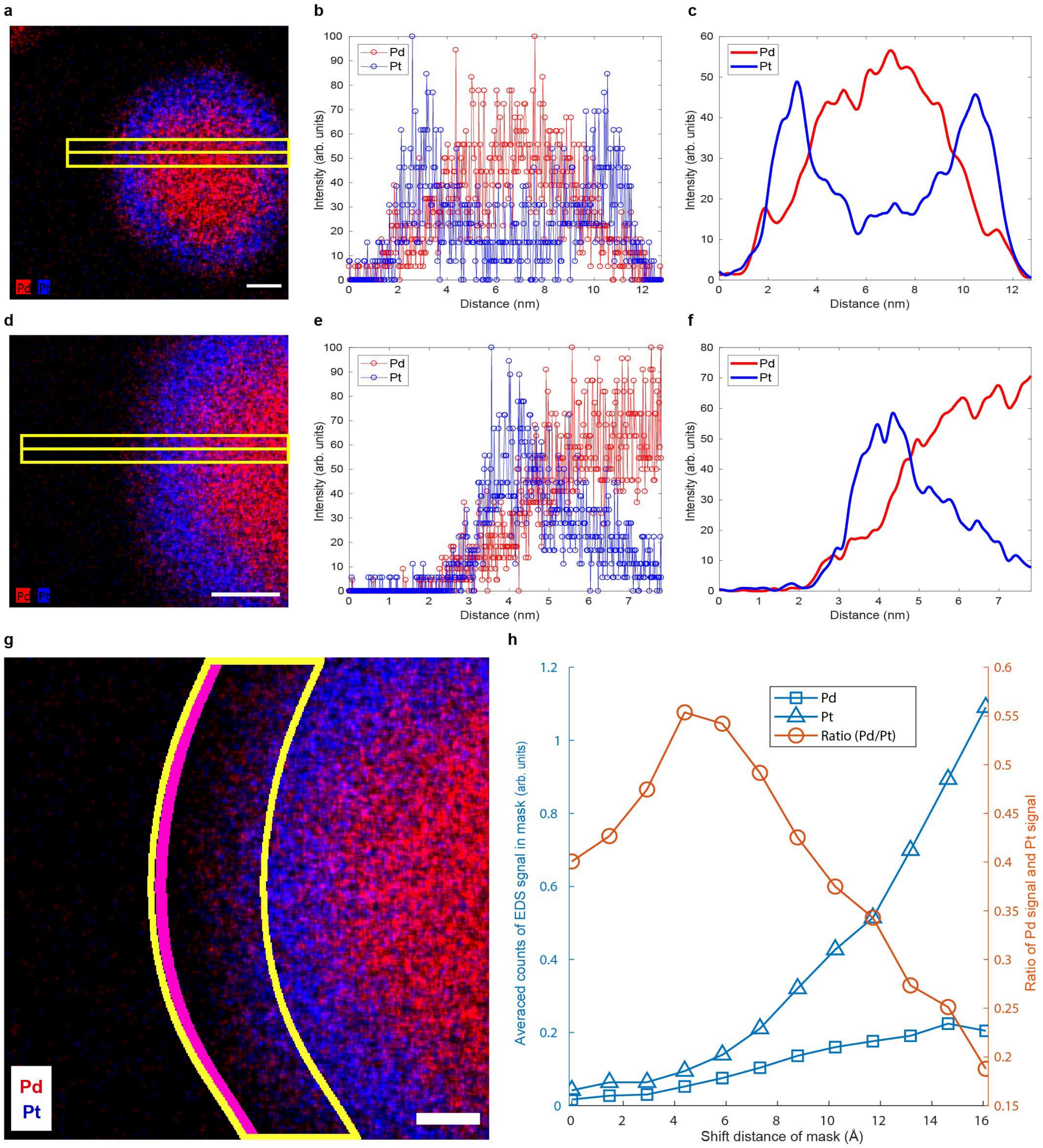

Supplementary Figure 1 | An elemental mapping image and line profiles from EDS experiments. a, An EDS elemental mapping image of a Pd@Pt nanoparticle. Scale bar, 2 nm. **b**, The raw intensity line scan profile within the yellow box of (**a**). **c**, The smoothed intensity line scan profile of (**b**) using a Gaussian kernel (σ = 1.94 Å). **d,** An EDS elemental mapping image of the Pd@Pt nanoparticle at higher magnification. Scale bar, 2 nm. **e**, The raw intensity line scan profile within the yellow box of (**d**). **f**, The smoothed intensity line scan profile of (**e**) using a Gaussian kernel (σ = 0.976 Å). **g,** A larger view of the EDS image shown in (**d**). The yellow line shows the region for the average intensity line scan profile in (**h**), and the magenta area represents the scanned mask within which the EDS signal was averaged to draw the line profile. Scale bar, 1 nm. **h**, The averaged intensity line scan profile within the region marked with a yellow line in (**g**). The triangle and square marks denote the average value of the EDS signals of the Pt and Pd atoms within the mask, respectively, and the circle marks represent the ratio of the average values from the two atomic species. A peak can be identified for the Pd/Pt signal ratio at the shell surface, evidencing the presence of the surface Pd within a few angstrom ranges. Note that the particle for this EDS analysis is a third particle, not Particle 1 or Particle 2 in the manuscript and that the data was smoothed (smoothing parameter 3 of ESPRIT Software from Bruker) without any background correction.

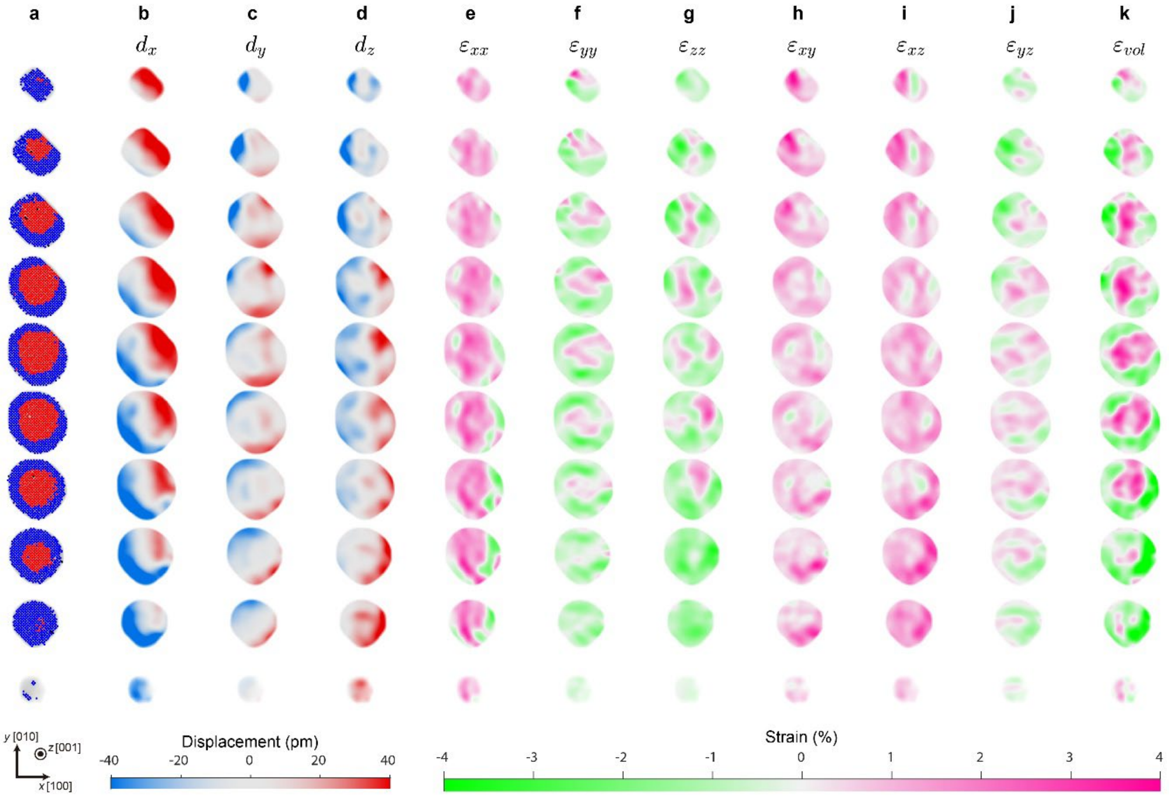

Supplementary Figure 2 | 3D atomic displacements and strain maps of the Pd@Pt nanoparticle in the Cartesian coordinate system (Particle 1). a, Atomic layers (sliced along the [001] direction) of the core-shell nanoparticle. Note that only one layer per every four atomic layers is plotted. Red and blue dots represent the positions of the Pd and Pt atoms assigned to the fcc lattice, respectively, and black dots represent the positions of atoms not assigned to the fcc lattice (Methods). **b-d,** 3D atomic displacement maps, along the x-axis (d_x) (**b**), along the y-axis (d_y) (**c**), and along the z-axis (d_z) (**d**), in the Cartesian coordinate system. **e-j,** 3D strain maps in Cartesian coordinates, representing principal components (**e-g**) and shear components (**h-j**) of the full strain tensor. **k,** The volumetric strain obtained by summing the three principal strain components. The atomic displacement and strain maps presented in (**b**-**k**) were calculated from the corresponding layers presented in (**a**).

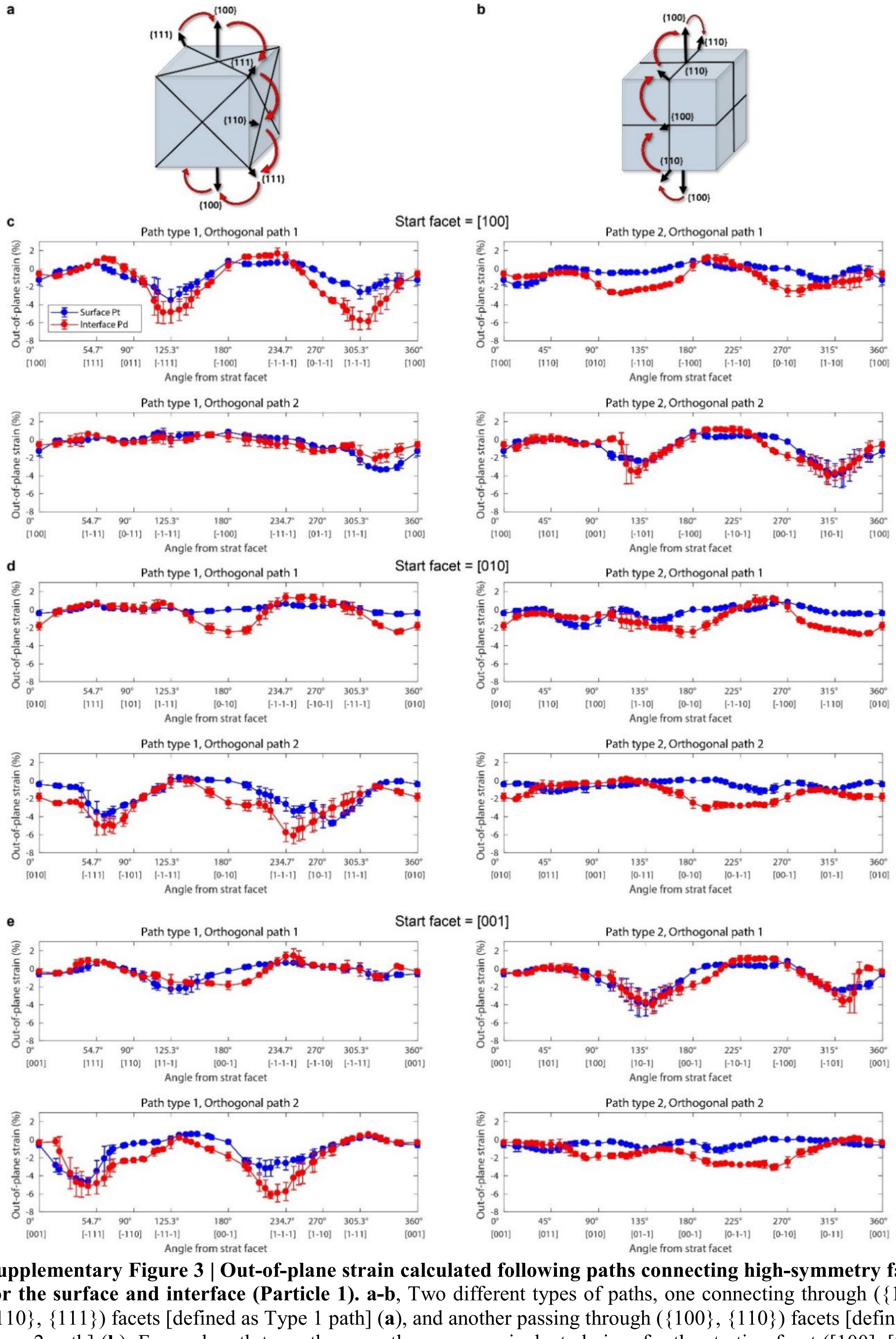

Supplementary Figure 3 | Out-of-plane strain calculated following paths connecting high-symmetry facets for the surface and interface (Particle 1). a-b, Two different types of paths, one connecting through ({100}, {110}, {111}) facets [defined as Type 1 path] (**a**), and another passing through ({100}, {110}) facets [defined as Type 2 path] (**b**). For each path type, there are three non-equivalent choices for the starting facet ([100], [010], and [001]), and two non-equivalent (orthogonal) paths are possible for each case, resulting in six different paths for each type. **c-e**, Calculated out-of-plane strains following paths connecting high-symmetry facets for the surface Pt and interface Pd. The starting facet directions are [100] (**c**), [010] (**d**), and [001] (**e**).

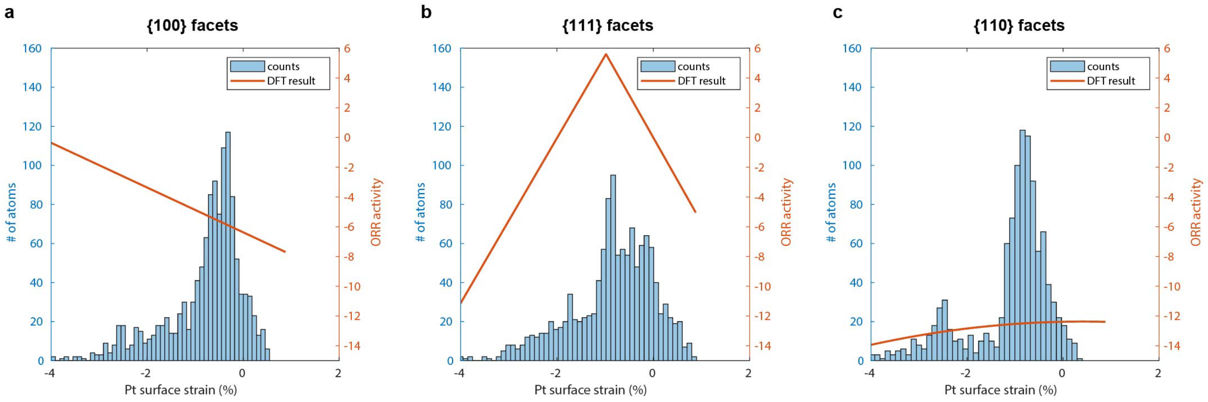

Supplementary Figure 4 | Strain-dependent ORR activity and histogram of surface Pt volumetric strain (Particle 1). a-c, The surface ORR activity as a function of the surface strain, obtained from DFT calculations[1–3] (orange lines), and histogram of the surface Pt volumetric strain, for {100} facets (**a**), {111} facets (**b**), and {110} facets (**c**), respectively. The ORR activity is represented as $\ln(j/j_{\mathrm{Pt},(111)})$ where j is the current density. Note that the Pd atoms on the exposed surface are not included in this analysis. The DFT result (orange lines) is provided to show the calculated relation between the ORR and local strain.

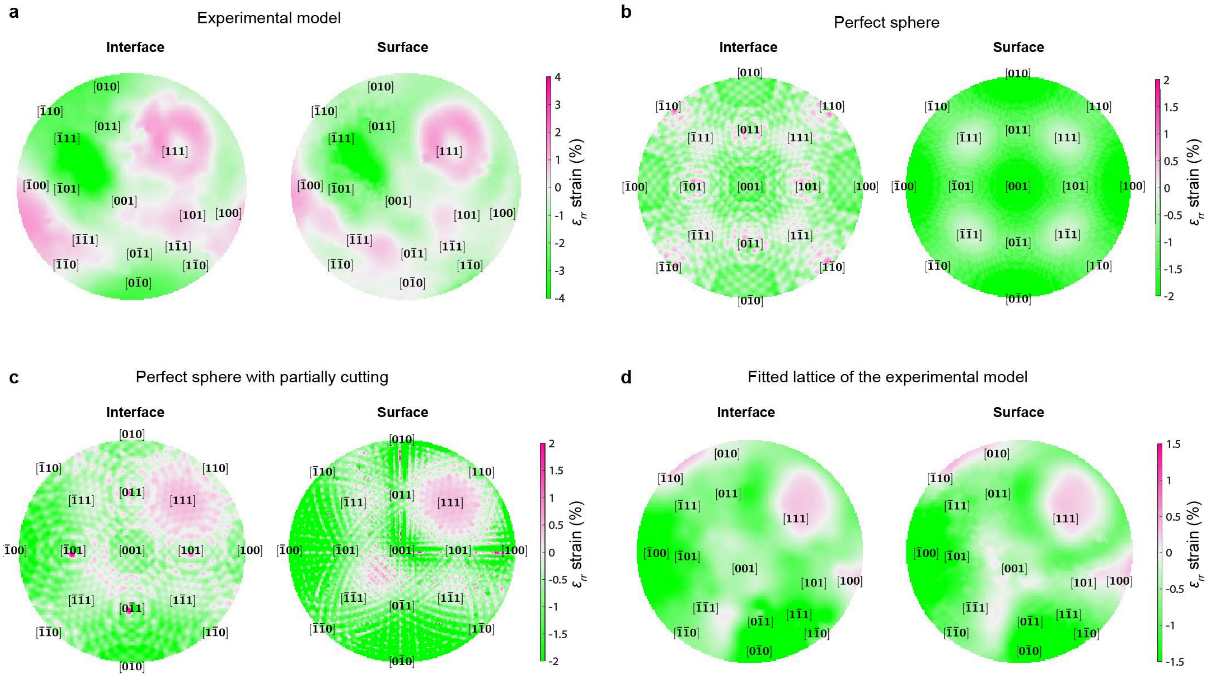

Supplementary Figure 5 | Surface and interface strain behaviors from the experimentally measured atomic structure and MS simulations of different nanoparticle shapes. a, The pole figures showing the interface and surface strains of the experimentally obtained atomic structure (Particle 1). **b-d,** The pole figures showing the interface and surface strains of the MS simulation results with the initial structure of a perfectly spherical shape (**b**), perfectly spherical shape with partial cutting (**c**), and the ideal fcc lattice model fitted to the experimental atomic structure of Particle 1 (**d**). The pole figures were obtained from stereographic projections of the radial strain at the interface and surface for the upper hemispheres. The Miller indices of low-index facets are marked either at the average position of the surface atoms assigned to each facet (Methods) (**a,d**), or at the corresponding mapping direction of stereographic projection (**b,c**).

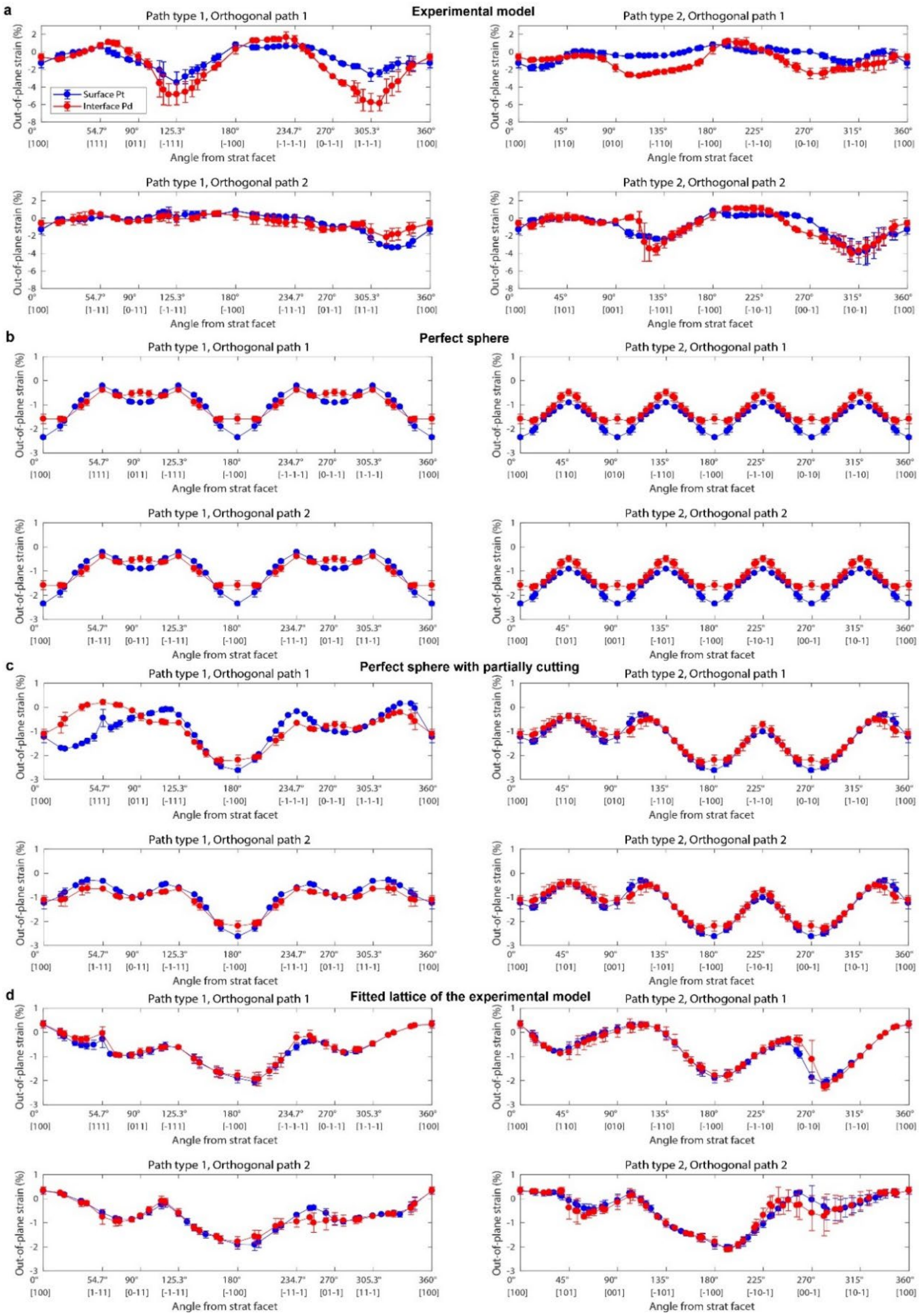

Supplementary Figure 6 | Surface and interface strains from the experimentally measured atomic structure and MS simulated structures of different nanoparticle shapes. a, Calculated out-of-plane strains of the experimentally obtained atomic structure (Particle 1) following paths connecting high-symmetry facets for the surface Pt and interface Pd. **b-d,** Calculated out-of-plane strains of the MS simulation results with the initial structure of a perfectly spherical shape (**b**), perfectly spherical shape with partial cutting (**c**), and the ideal fcc lattice model fitted to the experimental atomic structure of Particle 1 (**d**), following paths connecting high-symmetry facets for the surface Pt and interface Pd. The starting facet direction is [100]. Note that we used the path types defined in Supplementary Fig. 3.

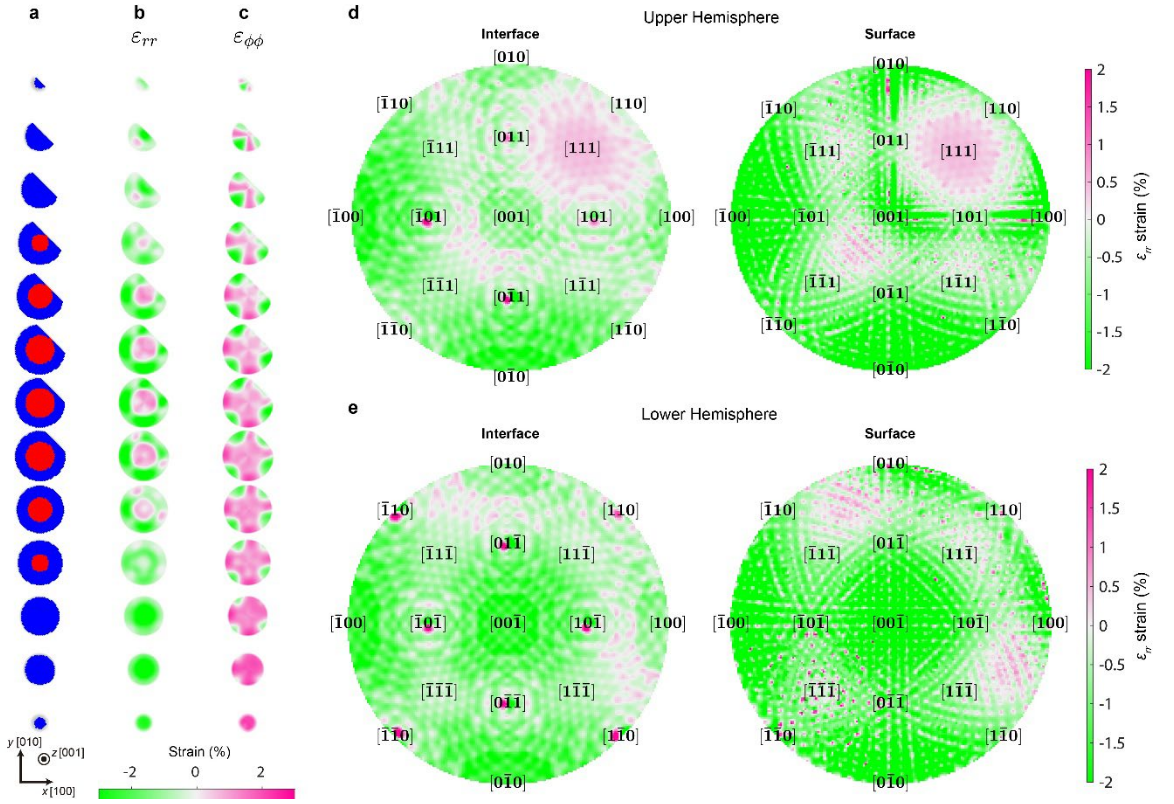

Supplementary Figure 7 | 3D strain maps and pole figures of an MS-simulated spherical core-shell structure with partial cutting. a, The MS-simulated spherical core-shell atomic structure, which was partially cut in order to model the exposed core part. The structure is divided into atomic layers along the [001] direction. Note that only one layer per every four atomic layers is plotted. The red and blue dots represent the positions of Pd and Pt atoms assigned to the fcc lattice, respectively. **b-c,** The calculated strain maps from the MS simulation result, for the radial (ε_{rr}) (**b**) and azimuthal ($\varepsilon_{\phi\phi}$) (**c**) strains in the spherical coordinate system, respectively. The strain maps were calculated from the corresponding layer presented in (**a**). **d-e,** Pole figures showing the interface and surface strains of the MS simulation result. The pole figures were obtained from stereographic projections of the radial strain at the interface and surface for the upper (**d**), and lower (**e**) hemispheres, respectively. The Miller indices of low-index facets are marked at the corresponding mapping direction of stereographic projection.

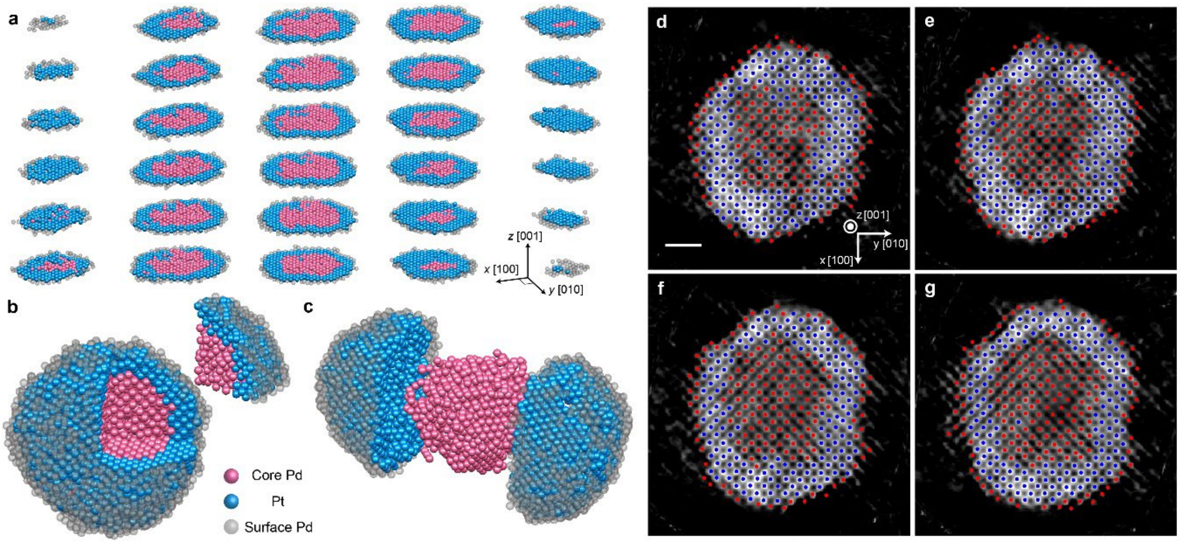

Supplementary Figure 8 | Experimentally determined 3D atomic structure of a Pd@Pt core-shell nanoparticle (Particle 2). a, Atomic layer slices along the [001] crystallographic direction, to show the overall core-shell structure. **b**, Overall 3D view of the nanoparticle with one octant of the sphere separated to visualize the core-shell architecture. **c**, Overall 3D structure with separated core and shell. The shell is cut in half showing the core and shell interface structure. **d-g**, 0.99 Å thick slices of the 3D tomogram, showing four consecutive atomic layers along the [001] direction, respectively. The grayscale background represents the intensity of the tomogram, and the red and blue dots represent the traced atomic coordinates of Pd and Pt, respectively. The Pd and Pt atoms can be well distinguished from the intensity contrast. Scale bar, 1 nm.

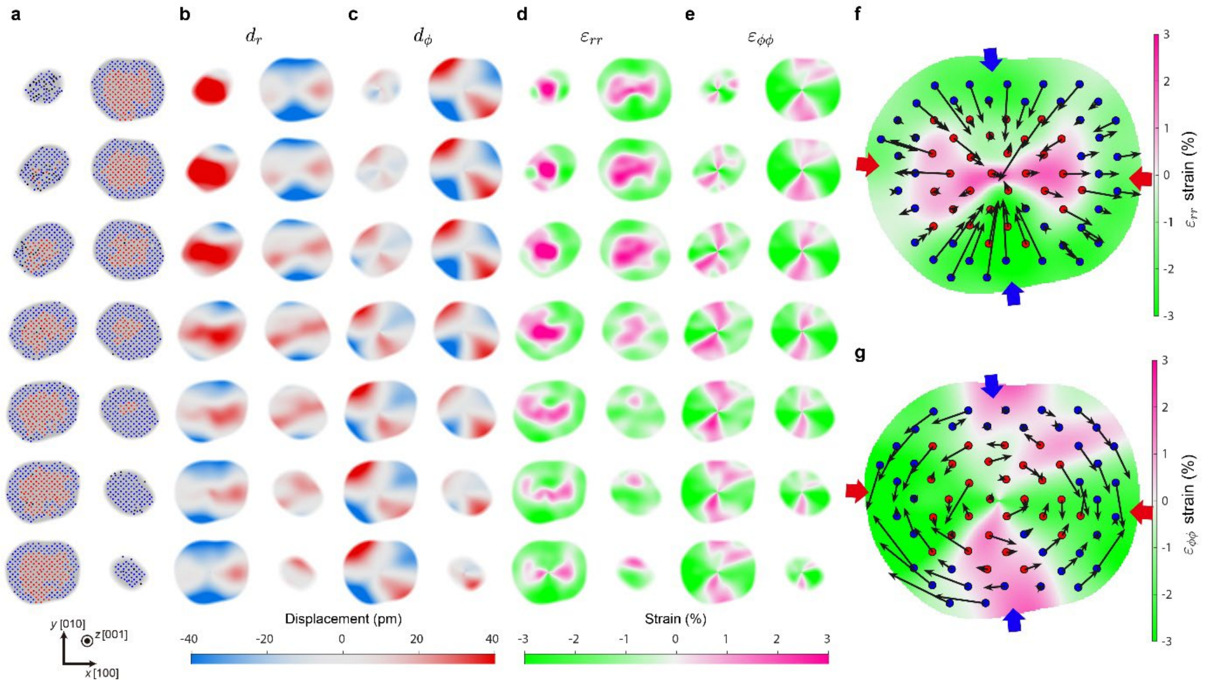

Supplementary Figure 9 | 3D atomic displacements and strain maps of the Pd@Pt nanoparticle (Particle 2). a, Atomic layers (sliced along the [001] direction) of the core-shell nanoparticle. Note that every atomic layer is plotted. Red and blue dots represent the positions of the Pd and Pt atoms assigned to the fcc lattice, respectively, and black dots represent the positions of atoms not assigned to the fcc lattice (Methods). **b-c**, 3D atomic displacement maps, along the radial direction (d_r) (**b**), and along the azimuthal direction (d_ϕ) (**c**) in the spherical coordinate system. **d-e,** 3D strain maps in spherical coordinates, representing radial (ε_{rr}) (**d**) and azimuthal ($\varepsilon_{\phi\phi}$) (**e**) strains, respectively (Methods). The atomic displacement and strain maps presented in (**b**-**e**) were calculated from the corresponding layer presented in (**a**). **f**, A map showing the 3D radial strain, atomic positions (only half of the atomic positions are plotted for better visualization), and radial displacement vectors of an atomic layer at the middle of the particle. **g**, Similar plot with (**f**), for azimuthal strain and displacements. Note that the radial displacements (d_r) point outward in the region where the azimuthal displacements (d_ϕ) converge in (the region pointed by red arrows), and the opposite behavior can be observed in the region where radial displacements point inward, where the azimuthal displacements diverge (the region pointed by blue arrows).

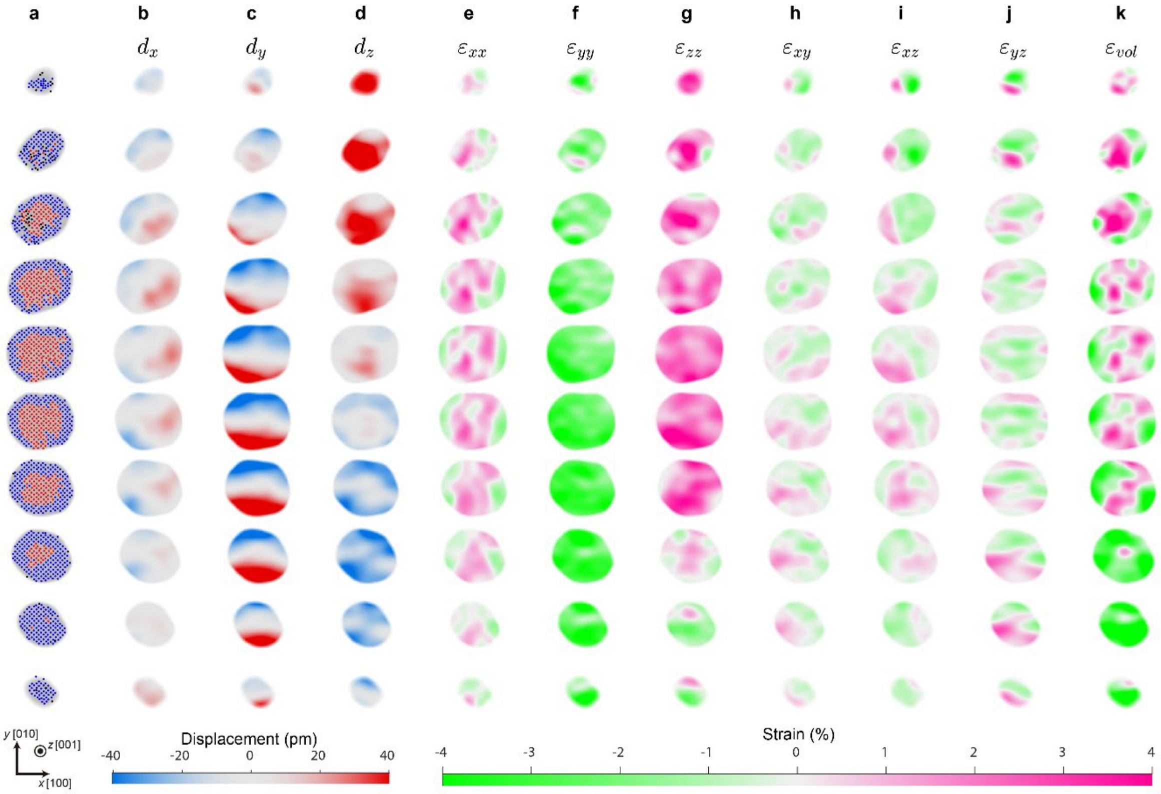

Supplementary Figure 10 | 3D atomic displacements and strain maps of the Pd@Pt nanoparticle in the Cartesian coordinate system (Particle 2). a, Atomic layers (sliced along the [001] direction) of the core-shell nanoparticle. Note that only one layer per every three atomic layers is plotted. Red and blue dots represent the positions of the Pd and Pt atoms assigned to the fcc lattice, respectively, and black dots represent the positions of atoms not assigned to the fcc lattice (Methods). **b-d,** 3D atomic displacement maps, along the x-axis (d_x) (**b**), along the y-axis (d_y) (**c**), and along the z-axis (d_z) (**d**), in the Cartesian coordinate system. **e-j,** 3D strain maps in Cartesian coordinates, representing principal components (**e-g**) and shear components (**h-j**) of the full strain tensor. **k,** The volumetric strain obtained by summing the three principal strain components. The atomic displacement and strain maps presented in (**b**-**k**) were calculated from the corresponding layers presented in (**a**).

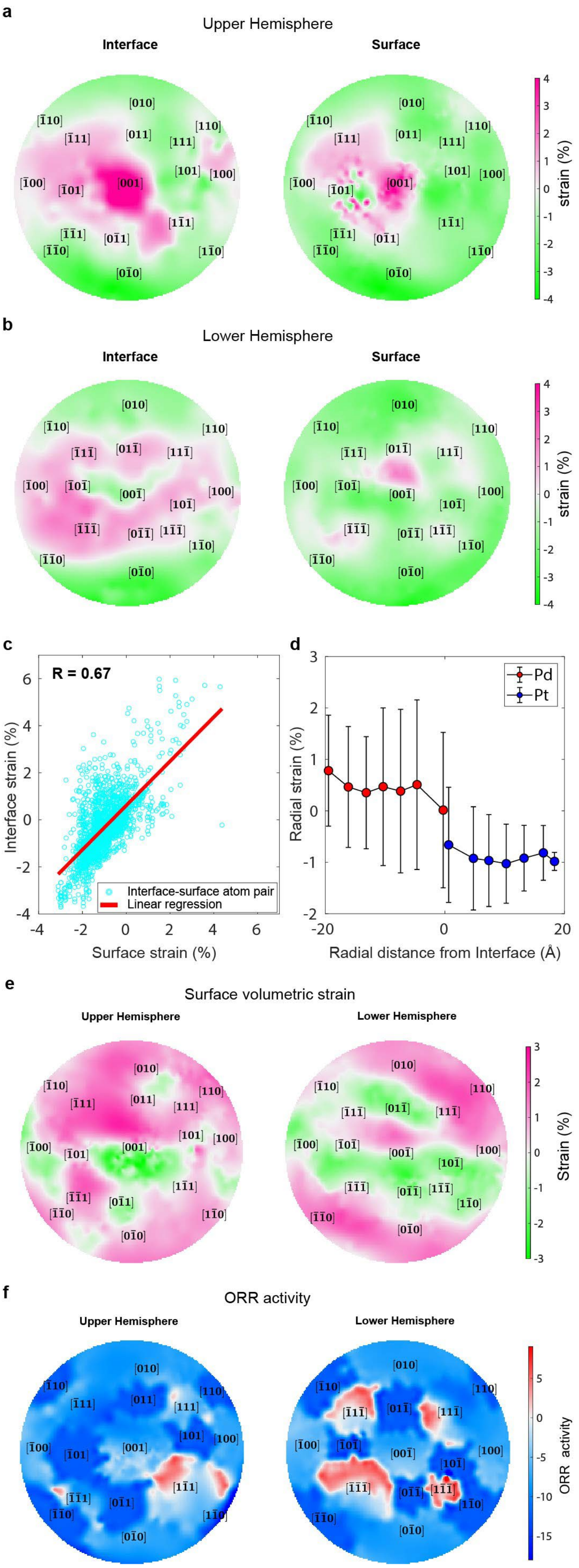

Supplementary Figure 11 | Surface and interface strain behavior, radial strain profile, and surface ORR activity (Particle 2). a-b, Pole figures obtained from stereographic projections of the radial strain at the interface and surface for the upper (**a**), and lower (**b**) hemispheres, respectively. The Miller indices of low-index facets are marked at the average position of the surface atoms assigned to each facet (Methods). **c**, A scatter plot showing the relation between the observed radial strains of surface and interface atoms. Each data point was obtained by paring each interface atom with the corresponding surface atom along the radial direction (Methods). A Pearson correlation coefficient (R) of 0.67 and slope of 0.94 were obtained from a linear regression (red line). **d**, The radial strain plotted as a function of the distance from the interface. Each data point and error bar represent an average and standard deviation of the strain values within the bins of 3 Å distance interval, respectively. **e**, Pole figures obtained by stereographic projections of the volumetric strain at the surface for the upper and lower hemispheres. **f**, Pole figures obtained from stereographic projections of the surface ORR activity for the upper and lower hemispheres. The ORR activity is represented as $\ln(j/j_{\mathrm{Pt},(111)})$ where j is the current density. The Miller indices of low-index facets are marked at the average position of the surface atoms assigned to each facet (Methods).

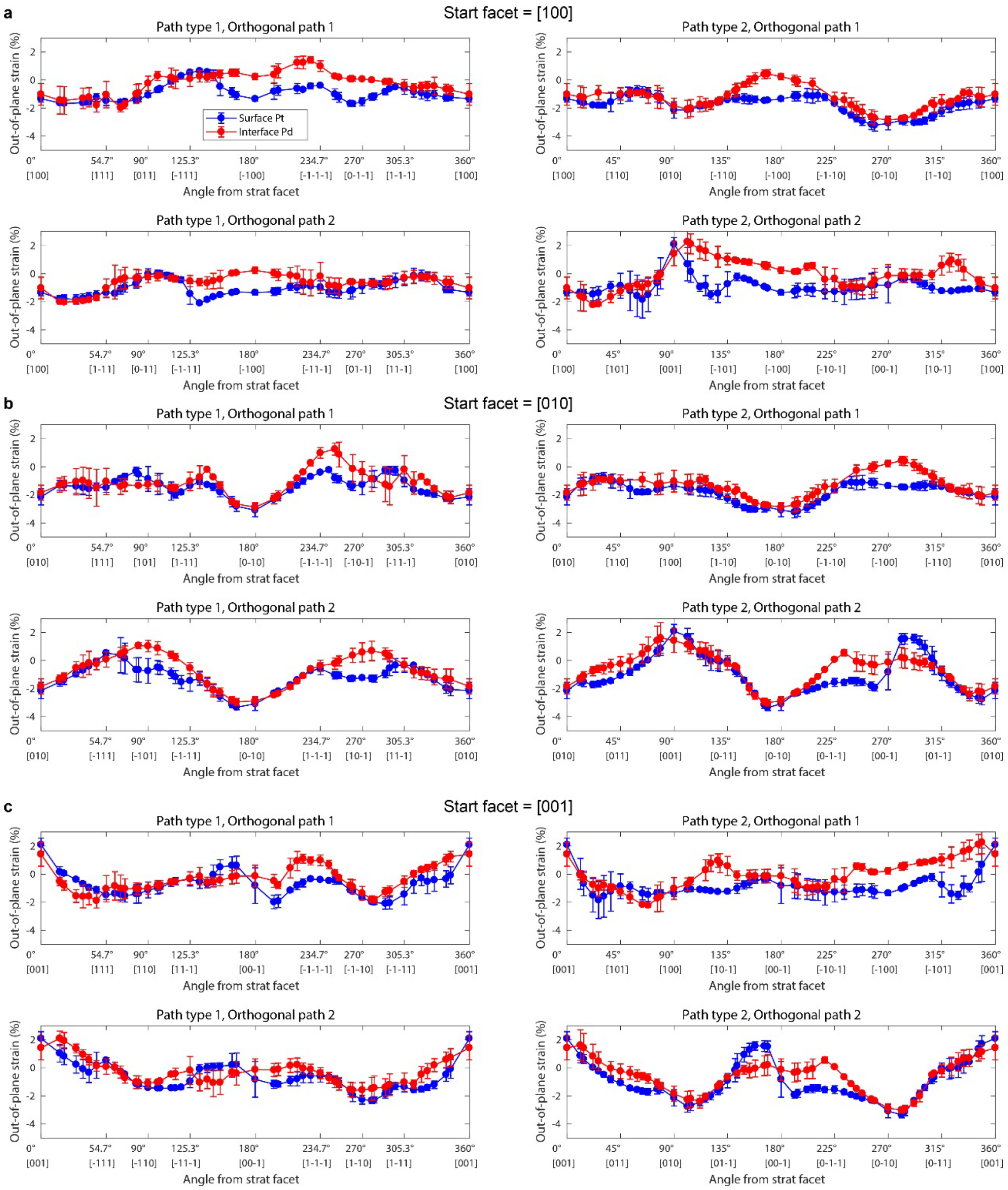

Supplementary Figure 12 | Out-of-plane strain calculated following paths connecting high-symmetry facets for the surface and interface (Particle 2). a-c, Calculated out-of-plane strains following paths connecting high-symmetry facets for the surface Pt and interface Pd. The starting facet directions are [100] (**a**), [010] (**b**), and [001] (**c**). Note that we used the path types defined in Supplementary Fig. 3.

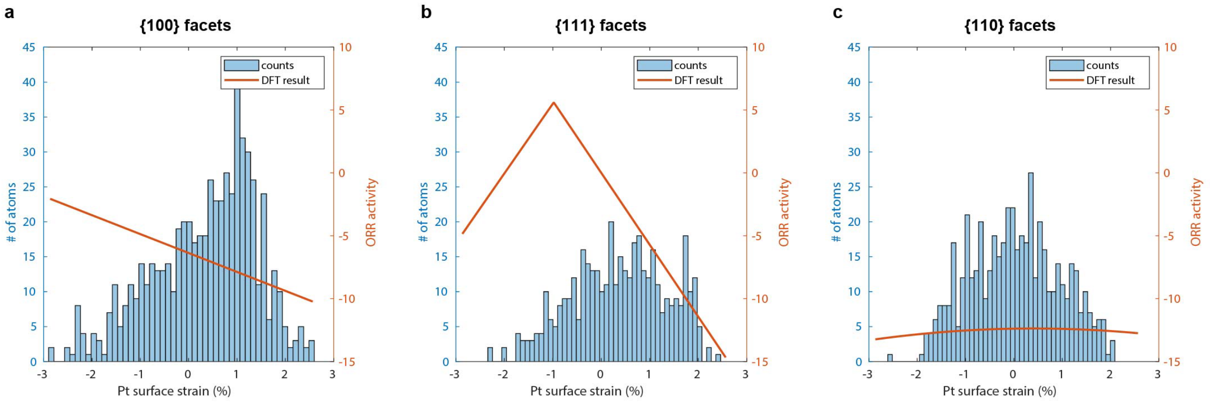

Supplementary Figure 13 | Strain-dependent ORR activity and histogram of surface Pt volumetric strain (Particle 2). a-c, The surface ORR activity as a function of the surface strain, obtained from DFT calculations[1–3] (orange lines), and histogram of the surface Pt volumetric strain, for $\{100\}$ facets (**a**), $\{111\}$ facets (**b**), and $\{110\}$ facets (**c**), respectively. The ORR activity is represented as $\ln(j/j_{\mathrm{Pt},(111)})$ where j is the current density. The DFT result (orange lines) is provided to show the calculated relation between the ORR and local strain.

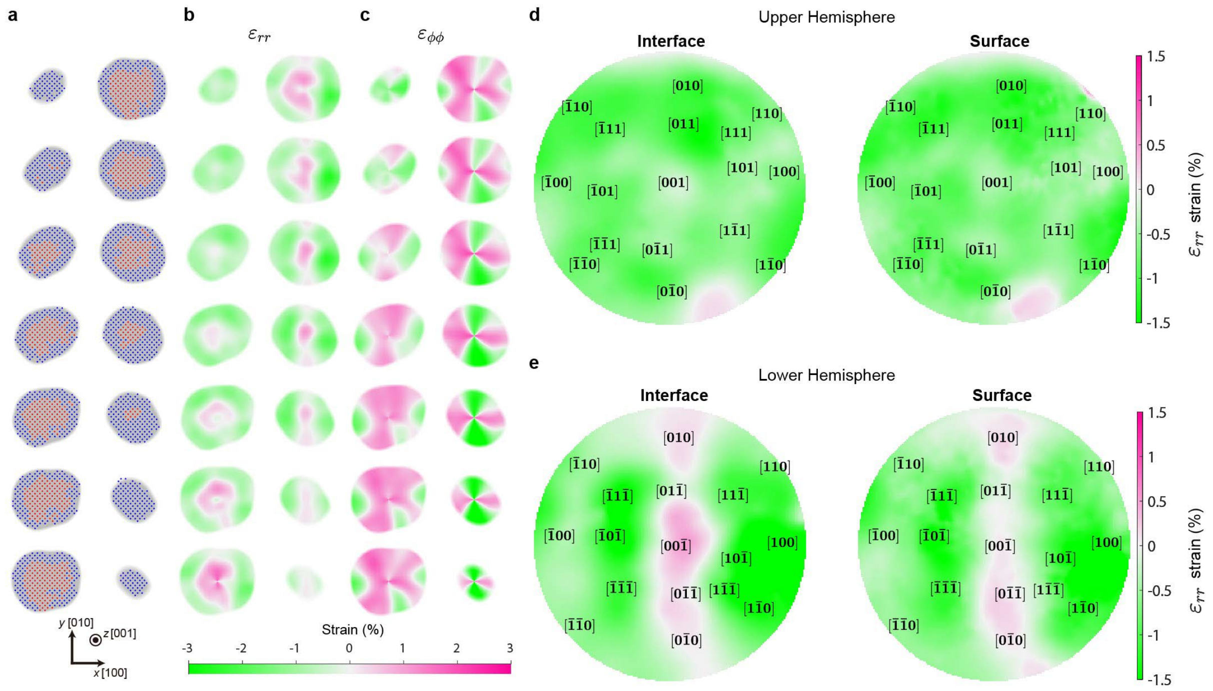

Supplementary Figure 14 | 3D strain maps and pole figures of the core-shell structure obtained by an MS simulation (Particle 2). a, The core-shell atomic structure resulting from the MS simulation (Methods), which is divided into atomic layers along the [001] direction. Note that every atomic layer is plotted. The red and blue dots represent the positions of Pd and Pt atoms assigned to the fcc lattice, respectively. **b-c,** The calculated strain maps from the MS simulation result for the radial (ε_{rr}) (**b**) and azimuthal ($\varepsilon_{\phi\phi}$) (**c**) strains in the spherical coordinate system, respectively. The strain maps were calculated from the corresponding layers presented in (**a**). **d-e,** Pole figures showing the interface and surface strains of the MS simulation result. The pole figures were obtained from stereographic projections of the radial strain at the interface and surface for the upper (**d**), and lower (**e**) hemispheres, respectively. The Miller indices of low-index facets are marked at the average position of the surface atoms assigned to each facet (Methods).

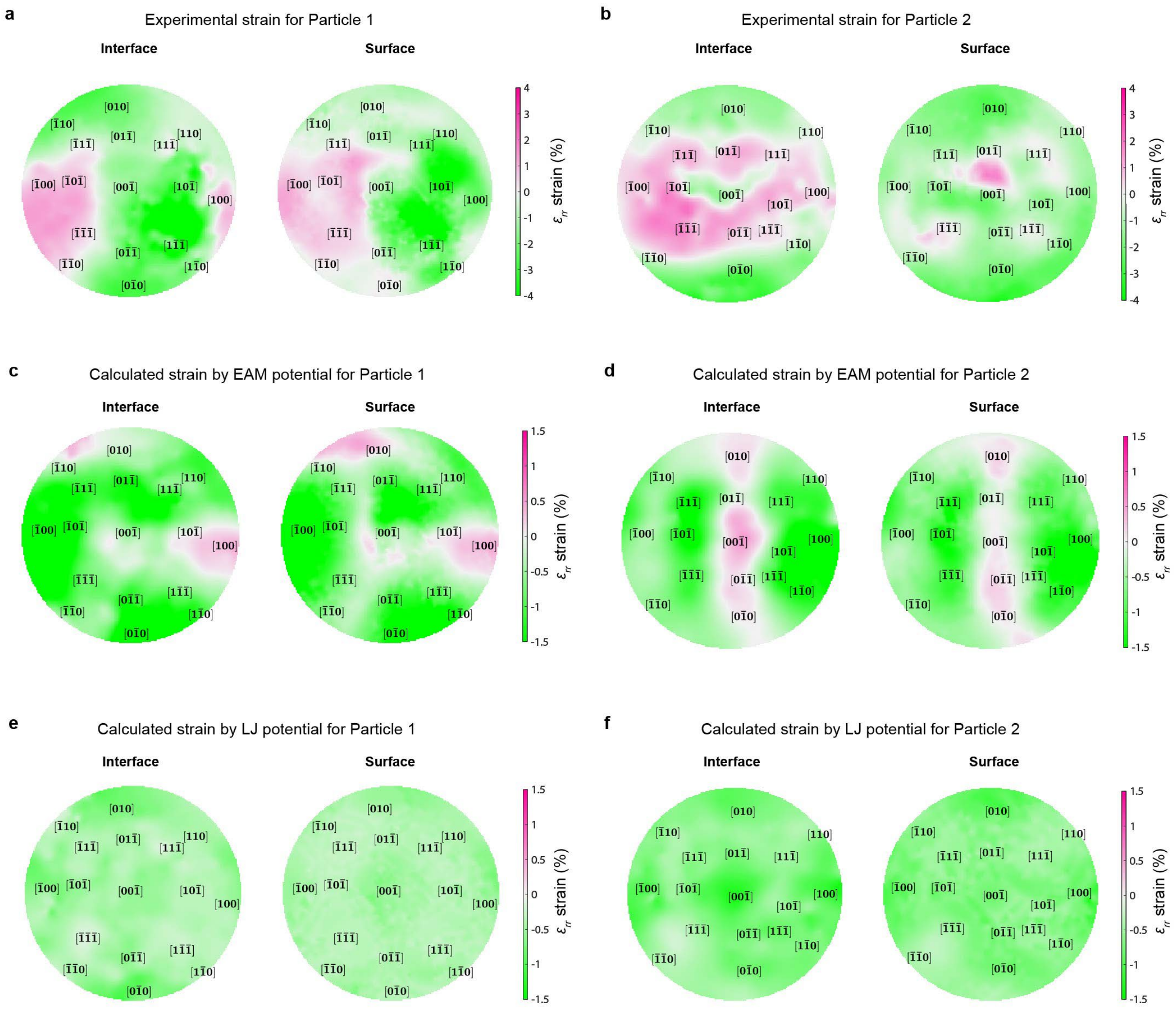

Supplementary Figure 15 | Surface and interface strain behaviors from the experimentally measured atomic structure and MS simulations which result from different interatomic potentials. a-b, The pole figures showing the interface and surface strains from the experimentally obtained atomic structure of Particle 1 (**a**), and Particle 2 (**b**). **c-d,** The pole figures showing the interface and surface strains from the MS simulation results with the ideal fcc lattice model fitted to the experimental atomic structure of Particle 1 (**c**), and Particle 2 (**d**) using the Ag-Al EAM potential (Methods). **e-f,** The pole figures showing the interface and surface strains from the MS simulation results with the ideal fcc lattice model fitted to the experimental atomic structure of Particle 1 (**e**), and Particle 2 (**f**) using an LJ potential (Methods). The pole figures were obtained from stereographic projections of the radial strain at the interface and surface for the lower hemispheres. The Miller indices of low-index facets are marked at the average position of the surface atoms assigned to each facet (Methods).

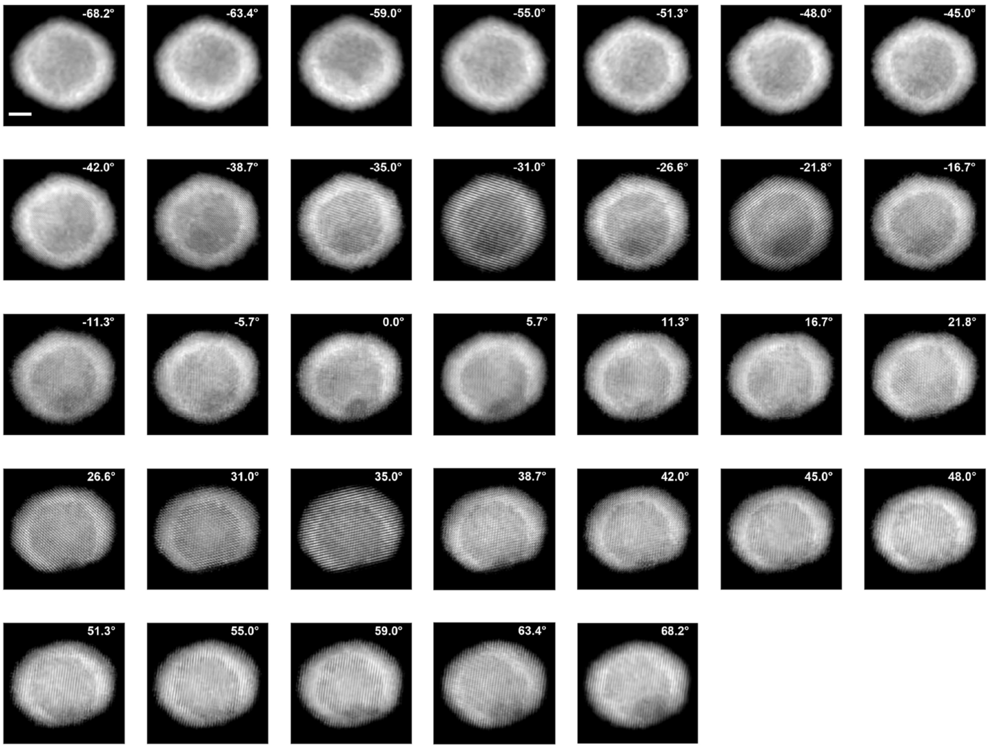

Supplementary Figure 16 | An experimental tomographic tilt series of the Pd@Pt nanoparticle (Particle 1). Total 33 tilt series images of Particle 1 were acquired from an ADF-STEM experiment and post-processed as described in the method section. The corresponding tilt angle for each projection is shown at the top right corner of each image. Scale bar, 2 nm.

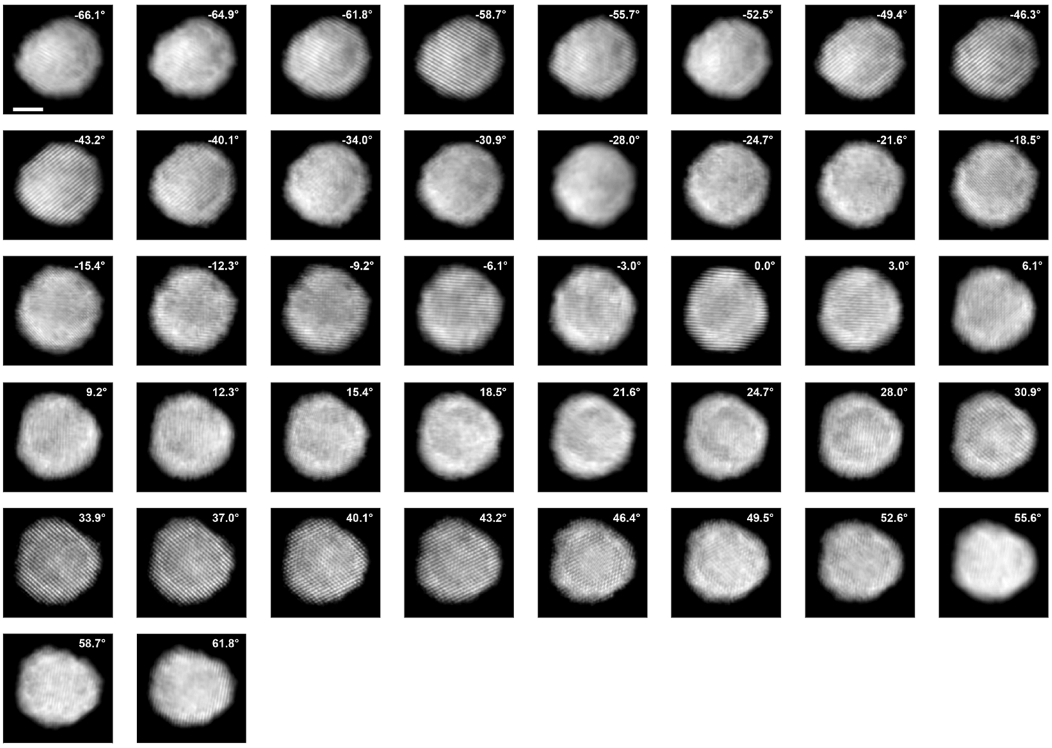

Supplementary Figure 17 | An experimental tomographic tilt series of the Pd@Pt nanoparticle (Particle 2). Total 42 tilt series images of Particle 2 were acquired from an ADF-STEM experiment and post-processed as described in the method section. The corresponding tilt angle for each projection is shown at the top right corner of each image. Scale bar, 2 nm.

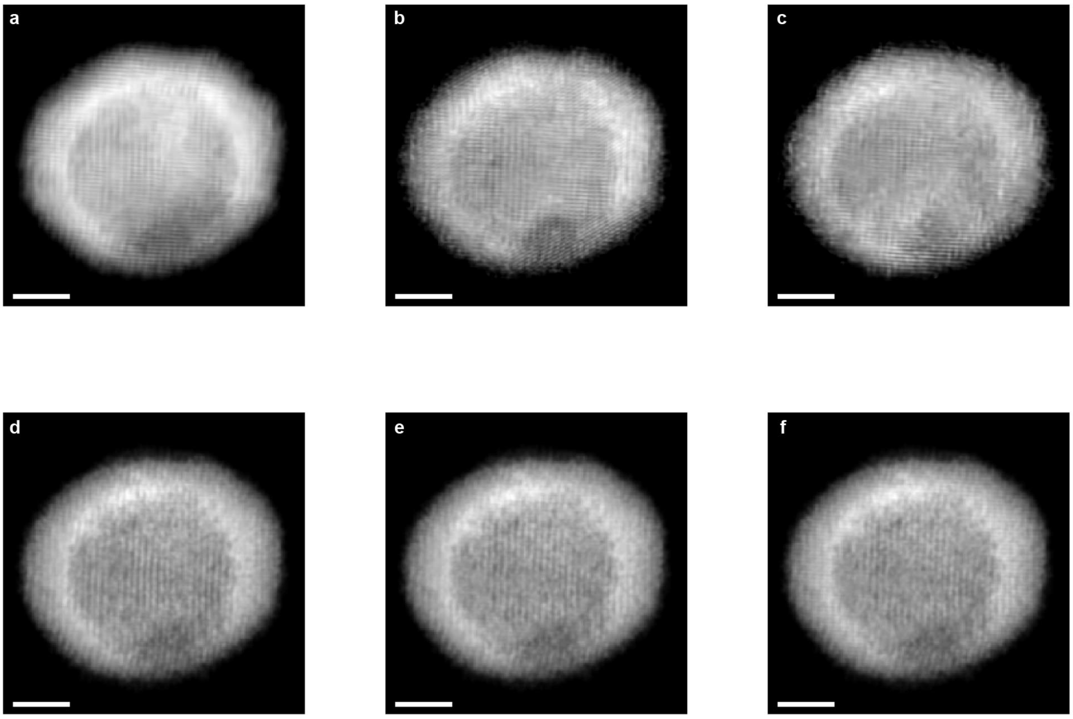

Supplementary Figure 18 | Experimental zero-degree projections and corresponding forward-projected projections from the final atomic model (Particle 1). a-c, Experimental zero-degree projections acquired at the beginning (**a**), in the middle (**b**), and at the end (**c**) of the experiment. The zero-degree projection of (**b**) was used for the reconstruction of the final 3D atomic model. **d-f**, Forward projections from the final 3D atomic model at the tilt angles which give the best consistency with the experimental zero-degree projections at the beginning (**d**), in the middle (**e**), and at the end (**f**) of the experiment (Methods). The best consistency angles were determined to be φ : 3.6°, θ : −1.0°, ψ : −0.4° (**d**), φ : −0.2°, θ : −0.2°, ψ : 0.6° (**e**), and φ : 0°, θ : −2.0°, ψ : −1.0° (**f**). Electron scattering factors of Pd and Pt atoms and Gaussian broadenings due to electron beam profile and thermal fluctuation (B-factors 6.07 Å^2 for Pd, 4.38 Å^2 for Pt) were considered for the forward projections. Scale bar, 2 nm.

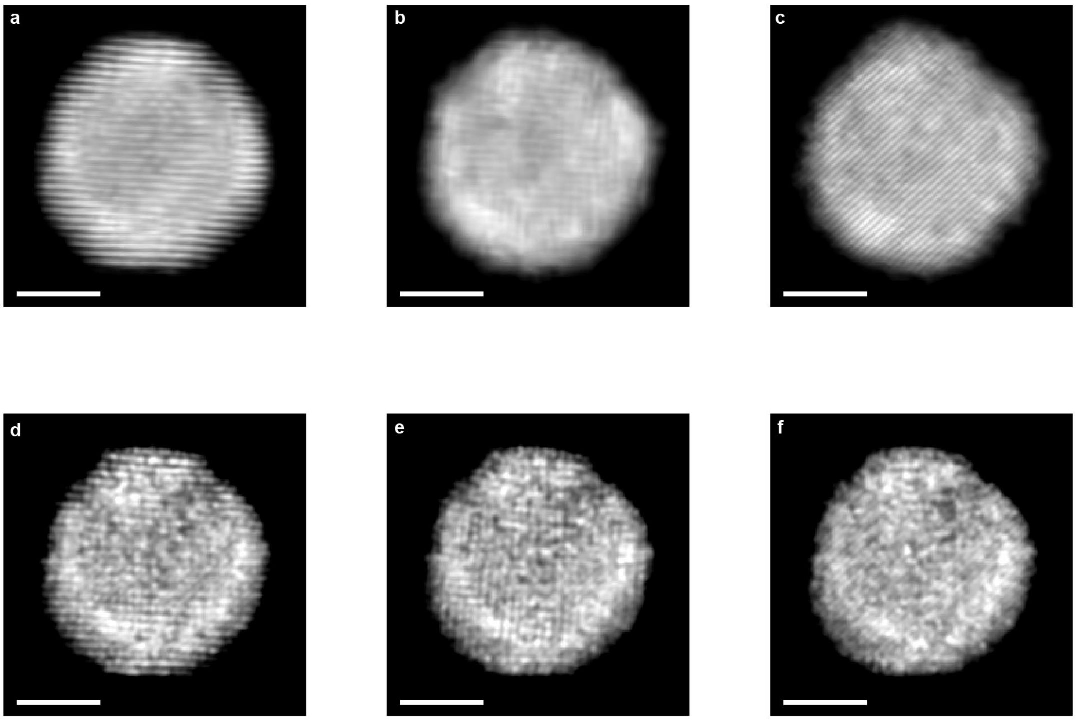

Supplementary Figure 19 | Experimental zero-degree projections and corresponding forward-projected projections from the final atomic model (Particle 2). a-c, Experimental zero-degree projections acquired at the beginning (**a**), in the middle (**b**), and at the end (**c**) of the experiment. The zero-degree projection of (**a**) was used for the reconstruction of the final 3D atomic model. **d-f**, Forward projections from the final 3D atomic model at the tilt angles which give the best consistency with the experimental zero-degree projections at the beginning (**d**), in the middle (**e**), and at the end (**f**) of the experiment (Methods). The best consistency angles were determined to be φ : 0.2°, θ : 0°, ψ : 1.6° (**d**), φ : 1.0°, θ : 4.4°, ψ : −1.0° (**e**), and φ : 0.2°, θ : 6.4°, ψ : 4.8° (**f**). Electron scattering factors of Pd and Pt atoms and Gaussian broadenings due to electron beam profile and thermal fluctuation (B-factors 8.57 Å^2 for Pd, 7.07 Å^2 for Pt) were considered for the forward projections. Scale bar, 2 nm.

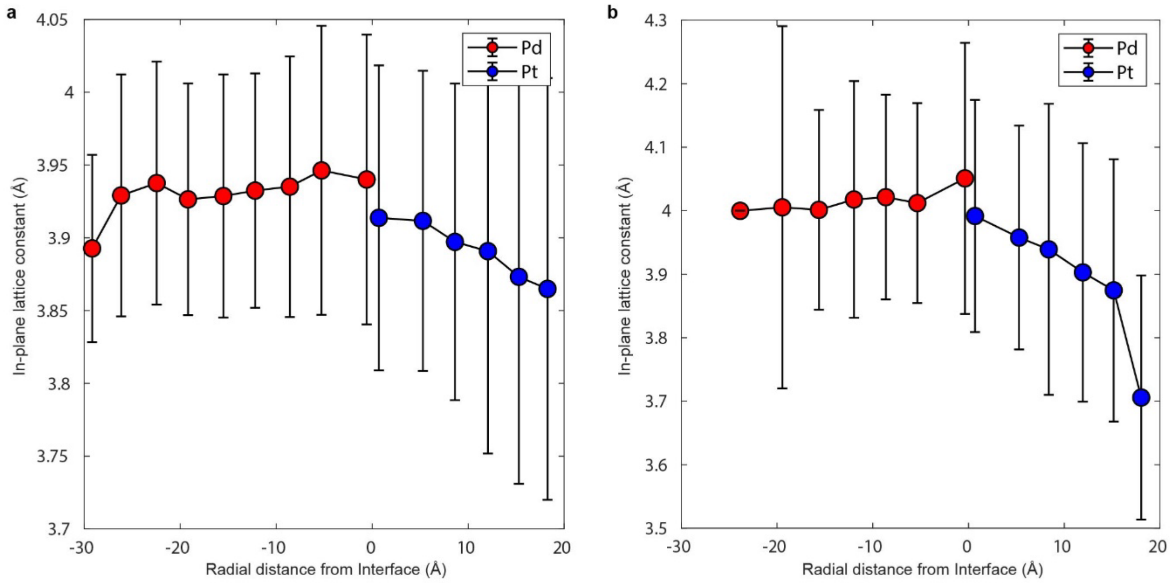

Supplementary Figure 20 | Radial profile of the in-plane lattice constant. a-b, The in-plane lattice constant plotted as a function of the distance from the interface for Particle 1 (**a**), and Particle 2 (**b**). Note that Particle 2 shows a relatively larger lattice constant at the interface (due to the relatively larger lattice constant of the Pd core for Particle 2), and also exhibits a faster rate of lattice relaxation compared to Particle 1. The in-plane lattice constants were estimated from the averaged in-plane nearest neighbor distance. The in-plane nearest neighbors for a given atom are defined as the nearest neighbor atoms for which the line connecting the given atom and the nearest neighbor atom makes an angle below 22.5° to the plane perpendicular to the position vector of the given atom. Each data point and error bar represent an average and standard deviation of the lattice constants within the bins of 3.5 Å interval, respectively.

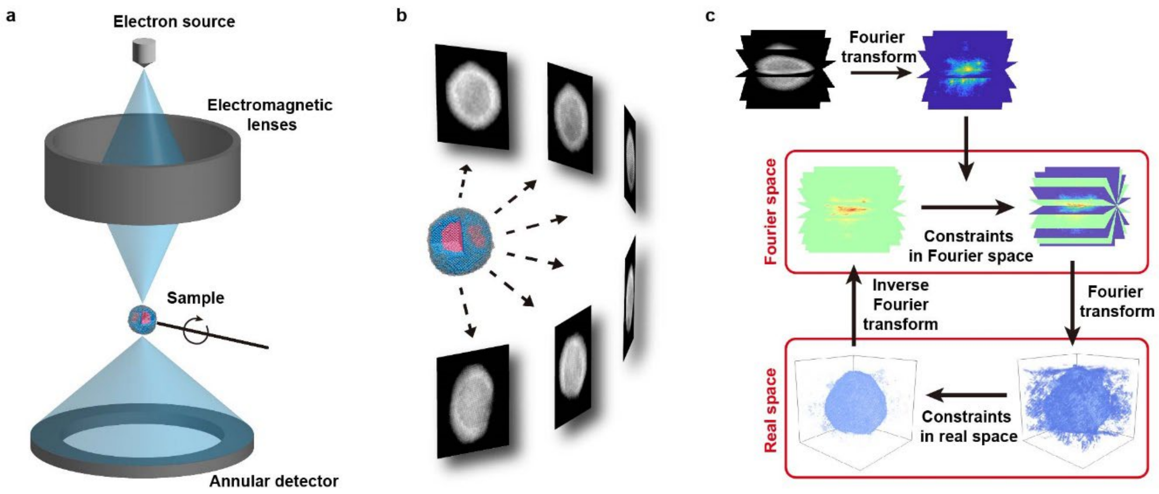

Supplementary Figure 21 | A schematic layout of atomic electron tomography based on ADF-STEM. a, An electron beam is focused on a small spot by electromagnetic lenses, and scanned over a specimen while the integrated signal at each scanning position is recorded by an annular detector to form a 2D projection image. **b**, A series of 2D projections are measured at different sample tilt angles. **c,** After image post-processing, the tilt series becomes converted to Fourier slices and a 3D reconstruction can be computed by using an iterative algorithm (for example, GENFIRE[4]).

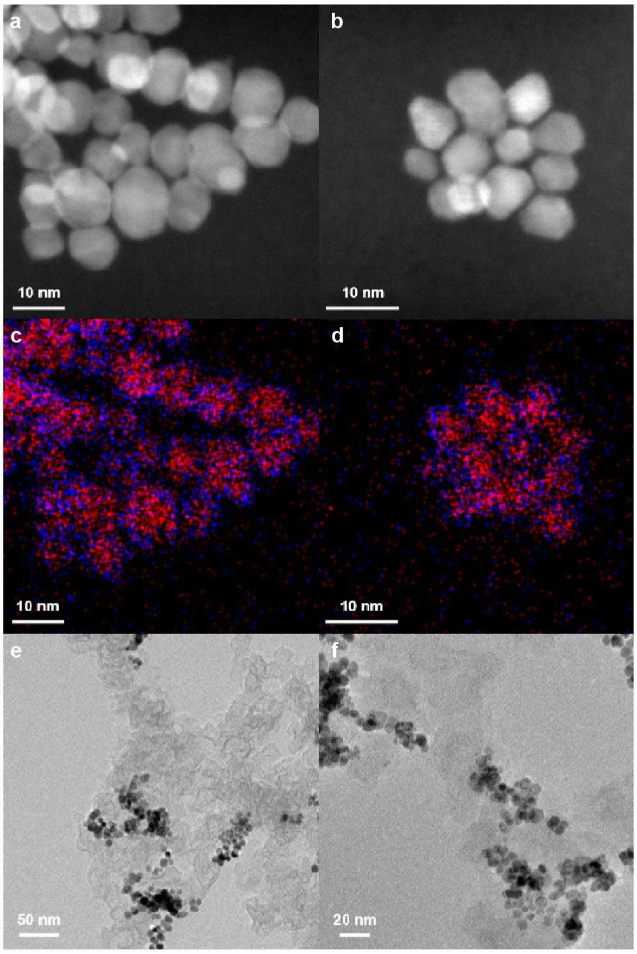

Supplementary Figure 22 | Structural and elemental analysis of newly synthesized samples for ORR measurements. a-b, ADF-STEM images of the two groups of synthesized Pd@Pt nanoparticles, for the group with the average diameter 8.56 nm [Sample 1] (**a**) and that with the average diameter 5.76 nm [Sample 2] (**b**). **c-d,** EDS elemental mapping images correspond to (**a**) and (**b**), respectively. **e-f**, Bright-field TEM images of the samples supported on a carbon black, for Sample 1 (**e**) and Sample 2 (**f**).

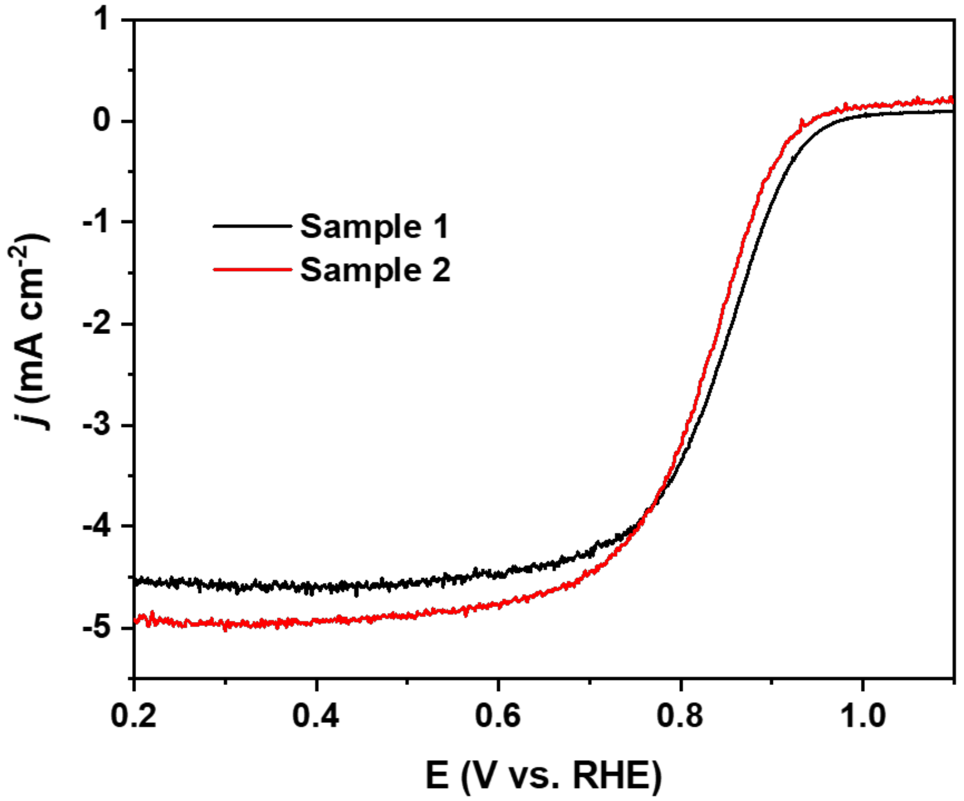

Supplementary Figure 23 | ORR polarization curves of samples in O_2-saturated 0.1 M $HClO_4$ at a scan rate of 10 mV s^{-1}.

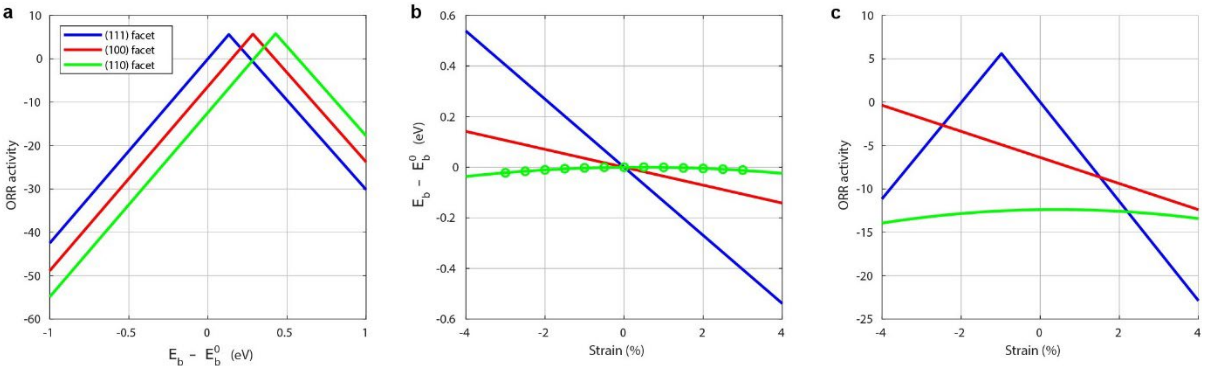

Supplementary Figure 24 | The relation between ORR activity, OH binding energy, and strain. a, Volcano-shaped relations between the ORR activity and OH binding energy for Pt {111}, {100} and {110} facets. The OH binding energy in the x-axis represents the relative change of the OH binding energy (E_b) with respect to the unstrained surfaces (E_b^0). **b,** Strain-dependent relative change of the OH binding energy for {111} and {100} facets (blue and red solid lines, respectively, obtained from a previous study[3]) and that for {110} facet (green circles; obtained from our own density functional theory calculation). The green solid line represents the fitted quadratic function for the green data points. **c,** The final relations between the ORR activity and strain for the three facet types obtained by combining the relations in (**a**) and (**b**). The ORR activity is represented as $\ln(j/j_{Pt,(111)})$ where j is the current density.

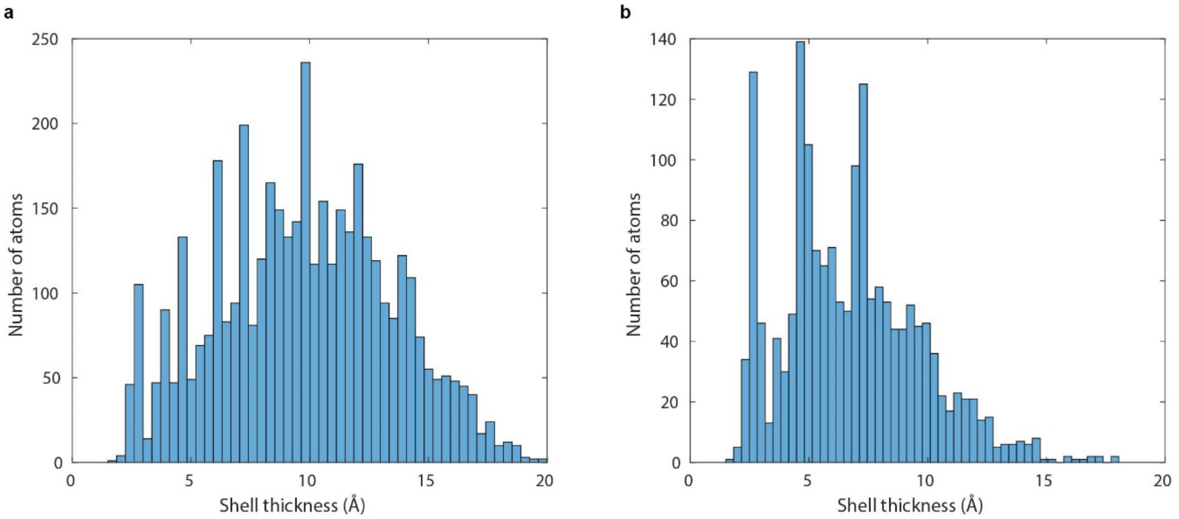

Supplementary Figure 25 | The histograms of the shell thickness determined from the interface-surface atom pair distances. a-b, Histogram of the shell thickness for Particle 1 (**a**), and Particle 2 (**b**). The average shell thickness values are 9.75 Å for Particle 1 and 6.77 Å for Particle 2.

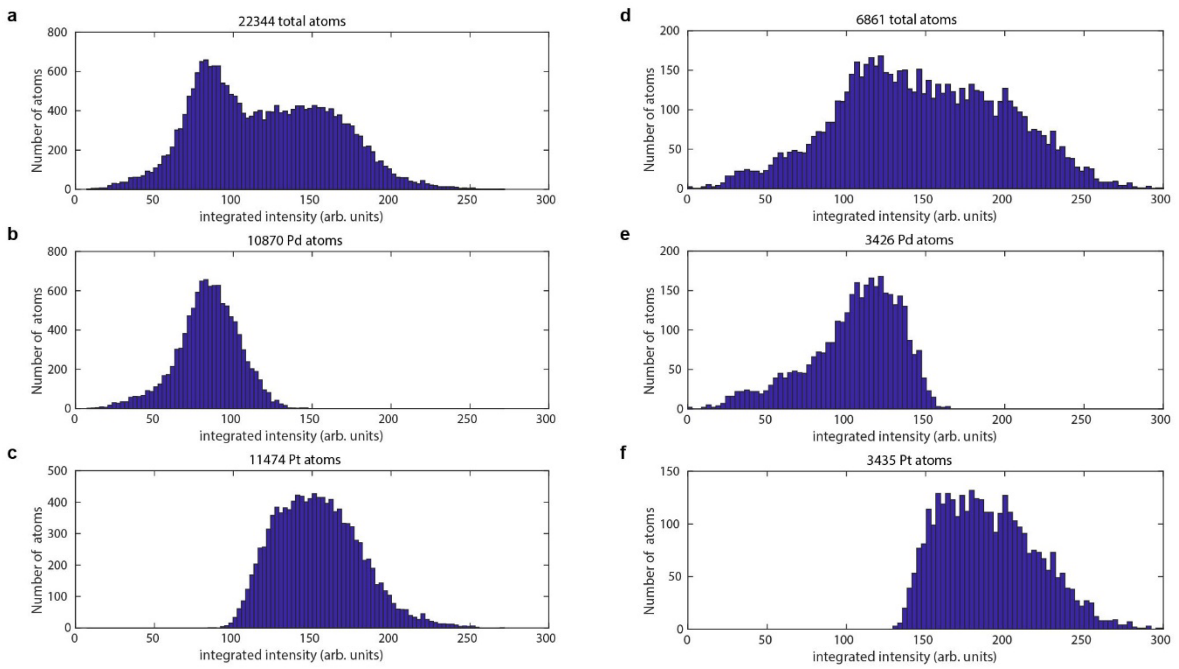

Supplementary Figure 26 | Classification of chemical species. **a-f**, Histograms of the local intensities of all traced atoms (**a,d**), those of the atoms classified as Pd (**b,e**), and those of the atoms classified as Pt (**c,f**) for Particle 1 (**a-c**), and Particle 2 (**d-f**).

Metal	Experiment		EAM	
	a_0(Å)	$A = \frac{2C_{44}}{C_{11} - C_{12}}$	a_0(Å)	$A = \frac{2C_{44}}{C_{11} - C_{12}}$
Pt	3.8749[a]	1.60[5]	3.98[6]	3.89[6]
PdH	3.9237[a]	2.08[7] 2.39[8]		
Pd			3.87[9]	3.05[9]
Al			4.05[10,11] 4.03[12]	1.25[10,11] 1.35[12]
Ni			3.52[9,11,13]	2.82[9,11,13]
Cu			3.62[12,13,14]	3.53[12] 3.23[13,13,15]
Ag			4.09[10,13,15]	3.00[10] 3.07[13,15]
Au			4.15[6] 4.08[13]	2.67[6] 2.90[13]

[a] Experiment result

Supplementary Table 1 | Lattice constants and Zener anisotropy ratio of FCC metals.

Supplementary References

1. Dietze, E. M. & Grönbeck, H. Structure-Dependent Strain Effects. *ChemPhysChem* **21**, 2407–2410 (2020).

2. Viswanathan, V., Hansen, H. A., Rossmeisl, J. & Nørskov, J. K. Universality in Oxygen Reduction Electrocatalysis on Metal Surfaces. *ACS Catal.* **2**, 1654–1660 (2012).

3. Lee, J., Jeong, C., Lee, T., Ryu, S. & Yang, Y. Direct Observation of Three-Dimensional Atomic Structure of Twinned Metallic Nanoparticles and Their Catalytic Properties. *Nano Lett.* **22**, 665–672 (2022).

4. Pryor, A. *et al.* GENFIRE: A generalized Fourier iterative reconstruction algorithm for high-resolution 3D imaging. *Sci. Rep.* **7**, 1–12 (2017).

5. Simmons, G. & Wang, H. *Single Crystal Elastic Constants and Calculated Aggregate Properties: A Handbook*. (MIT Press, 1971).

6. O'Brien, C. J., Barr, C. M., Price, P. M., Hattar, K. & Foiles, S. M. Grain boundary phase transformations in PtAu and relevance to thermal stabilization of bulk nanocrystalline metals. *J. Mater. Sci.* **53**, 2911–2927 (2018).

7. Nygren, L. A. & Leisure, R. G. Elastic constants of α'-phase PdHx over the temperature range 4-300 K. *Phys. Rev. B* **37**, 6482–6485 (1988).

8. Hsu, D. K. & Leisure, R. G. Elastic constants of palladium and β-phase palladium hydride between 4 and 300 K. *Phys. Rev. B* **20**, 1339–1344 (1979).

9. Samolyuk, G. D., Béland, L. K., Stocks, G. M. & Stoller, R. E. Electron–phonon coupling in Ni-based binary alloys with application to displacement cascade modeling. *J. Phys. Condens. Matter* **28**, 175501 (2016).

10. AlAg - EAM potentials. https://sites.google.com/site/eampotentials/AlAg.

11. Purja Pun, G. P. & Mishin, Y. Development of an interatomic potential for the Ni-Al system. *Philos. Mag.* **89**, 3245–3267 (2009).

12. Liu, X.-Y., Liu, C.-L. & Borucki, L. J. A new investigation of copper's role in enhancing Al–Cu interconnect electromigration resistance from an atomistic view. *Acta Mater.* **47**, 3227–3231 (1999).

13. Fischer, F., Schmitz, G. & Eich, S. M. A systematic study of grain boundary segregation and grain boundary formation energy using a new copper–nickel embedded-atom potential. *Acta Mater.* **176**, 220–231 (2019).

14. Zhou, X. W., Johnson, R. A. & Wadley, H. N. G. Misfit-energy-increasing dislocations in vapor-deposited CoFe/NiFe multilayers. *Phys. Rev. B* **69**, 144113 (2004).

15. Williams, P. L., Mishin, Y. & Hamilton, J. C. An embedded-atom potential for the Cu–Ag system. *Model. Simul. Mater. Sci. Eng.* **14**, 817–833 (2006).
