# Supplementary material for: Direct strain correlations at the single-atom level in three-dimensional core-shell interface structures
Source: Nat Commun. 2022 Oct 10;13:5957. doi: 10.1038/s41467-022-33236-6 (PMC9551052; doi:10.1038/s41467-022-33236-6)
Supplement: Supplementary file 3 — Description of Additional Supplementary Information [file 41467_2022_33236_MOESM3_ESM.pdf]

Description of Additional Supplementary Information

Title: Supplementary Movie 1 |

Description: Intensity and traced atom position maps of the Pd@Pt nanoparticle (Particle 1). 1.06 Å thick slices of the 3D tomogram of Particle 1, showing all atomic layers along the [001] direction. Grayscale background represents the intensity of the tomogram, and the red and blue dots represent the traced atomic coordinates of Pd and Pt, respectively. Scale bar, 1 nm.

Title: Supplementary Movie 2 |

Description: Traced atom positions and assigned ideal fcc lattice maps of the Pd@Pt nanoparticle (Particle 1). All atomic layers (sliced along the [001] direction) of Particle 1. Red and blue dots represent the positions of the Pd and Pt atoms assigned to the fcc lattice, respectively, and black dots represent the positions of atoms not assigned to the fcc lattice. Hollow green circles represent the positions of the assigned ideal fcc lattice sites.

Title: Supplementary Movie 3 |

Description: Radial strains at the interface and surface of the Pd@Pt nanoparticle (Particle 1). The movie shows the Pd interface and Pt surface atomic structure, where the radial strains are plotted at each atomic position. The linear color map indicates the radial strain from -2% (minimum) to +2% (maximum). The diameter of the circle representing each atom is 1.99 Å

Title: Supplementary Movie 4 |

Description: Assigned facet, local lattice constant, surface strain, and surface ORR activity of the Pd@Pt nanoparticle (Particle 1). a, Surface Pt atoms which are classified into the three dominant facet families of {111}, {100} and {110}. Red, green and blue dots represent the atom positions that are assigned to {100}, {111} and {110}, respectively. **b**, The atomic structure of the surface where the kernel averaged local lattice constants are plotted at each atomic position. The linear color map indicates the local lattice constant from 3.75 Å (minimum) to 3.95 Å (maximum). **c**, The atomic structure of the surface where the volumetric strains are plotted at each atomic position. The linear color map indicates the volumetric strain from -3% (minimum) to +3% (maximum). **d**, The atomic structure of the surface where the ORR activities are plotted at each atomic position. The linear color map indicates the ORR activity from -16 to 8, and the ORR activity is represented as $\ln(j/j_{\mathrm{Pt},(111)})$ where j is the current density.

Title: Supplementary Movie 5 |

Description: Intensity and traced atom position maps of the Pd@Pt nanoparticle (Particle 2). 0.99 Å thick slices of the 3D tomogram of Particle 2, showing all atomic layers along the [001] direction. Grayscale background represents the intensity of the tomogram, and the red and blue dots represent the traced atomic coordinates of Pd and Pt, respectively. Scale bar, 1 nm.

Title: Supplementary Movie 6 |

Description: Traced atom positions and assigned ideal fcc lattice maps of the Pd@Pt nanoparticle (Particle 2). All atomic layers (sliced along the [001] direction) of Particle 2. Red and blue dots represent the positions of the Pd and Pt atoms assigned to the fcc lattice, respectively, and black dots represent the positions of atoms not assigned to the fcc lattice. Hollow green circles represent the positions of the assigned ideal fcc lattice sites.

Title: Supplementary Movie 7 |

Description: Radial strains at the interface and surface of the Pd@Pt nanoparticle (Particle 2). The movie shows the Pd interface and Pt surface atomic structure, where the radial strains are plotted at each atomic position. The linear color map indicates the radial strain from -3% (minimum) to +3% (maximum). The diameter of the circle representing each atom is 2.21 Å

Title: Supplementary Movie 8 |

Description: Assigned facet, local lattice constant, surface strain, and surface ORR activity of the Pd@Pt nanoparticle (Particle 2). a, Surface Pt atoms which are classified into the three dominant facet families of {111}, {100} and {110}. Red, green and blue dots represent the atom positions that are assigned to {100}, {111} and {110}, respectively. **b**, The atomic structure of the surface where the kernel averaged local lattice constants are plotted at each atomic position. The linear color map indicates the local lattice constant from 3.80 Å (minimum) to 4.00 Å (maximum). **c**, The atomic structure of the surface where the volumetric strains are plotted at each atomic position. The linear color map indicates the volumetric strain from -3% (minimum) to +3% (maximum). **d**, The atomic structure of the surface where the ORR activities are plotted at each atomic position. The linear color map indicates the ORR activity from -16 to 8, and the ORR activity is represented as $\ln(j/j_{\mathrm{Pt},(111)})$ where j is the current density.
