# Supplementary material for: Direct strain correlations at the single-atom level in three-dimensional core-shell interface structures
Source: Nat Commun. 2022 Oct 10;13:5957. doi: 10.1038/s41467-022-33236-6 (PMC9551052; doi:10.1038/s41467-022-33236-6)
Supplement: Supplementary file 13 — Source Data [file 41467_2022_33236_MOESM13_ESM.zip › README.pdf]

1) SourceData_Fig3c.xlsx : Source data of Figure 3c

- First column : Radial strain of paired surface atoms (x-axis data)
- Second column : Radial strain of interface atoms (y-axis data)
- Third column : Intercept (first row) and slope (second row) of linear regression result

2) SourceData_Fig3d.xlsx : Source data of Figure 3d

- First column : Radial distance from interface for each atom (x-axis data)
- Second column : Radial strain for each atom (y-axis data)
- Third column : Atomic species for each atom (1: Pd, 2: Pt)
